# Retinal waves are unlikely to instruct the formation of eye-specific retinogeniculate projections

**DOI:** 10.1186/1749-8104-4-25

**Published:** 2009-07-06

**Authors:** Leo M Chalupa

**Affiliations:** 1 Department of Neurobiology, Physiology and Behavior, College of Biological Sciences, and Department of Ophthalmology and Vision Science, School of Medicine, University of California, Davis, CA 95616, USA, and Office of the Vice President for Research, The George Washington University, Washington DC 20052

## Abstract

In all mammalian species the projections of the two eyes to the dorsal lateral geniculate nucleus are initially overlapping before gradually forming the eye-specific domains evident at maturity. It is widely thought that retinal waves of neuronal activity play an instructional role in this developmental process. Here, I discuss the myriad reasons why retinal waves are unlikely to have such a role, and suggest that eye-specific molecular cues in combination with neuronal activity are most probably involved in the formation of eye-specific retinogeniculate projections.

## Introduction

To observe retinal waves is a wondrous thing. A freshly removed piece of retinal tissue from a developing animal is placed in a recording chamber containing an array of microelectrodes. Within a few minutes there appears a burst of activity at one of the electrodes that spreads in a wave-like fashion to adjoining recording sites, before dissipating to a quiescent state. Things remain dormant for what seems like an excruciatingly long interval before another burst of action potentials appears, traveling across the retinal surface with a different propagation pattern. This scenario can continue for many hours as long as the retinal specimen remains viable.

In the past decade or so, retinal waves have been the darling topic of developmental visual neurobiologists. The main reason for this is that the presence of such correlated discharge patterns provides a plausible and parsimonious mechanism by which neuronal activity could act to refine the architecture of ganglion cell dendrites as well as the early imprecise patterns of retinal projections [1,2]. Such activity would seem to be ideally suited for fulfilling one of the key requirements of the famous Hebbian postulate that cells that fire together wire together [3].

I wish to raise caution about the widely prevalent notion that retinal waves play an instructional role in the formation of segregated eye-specific retinogeniculate projections. This refers to the fact that in all mammalian species retinal ganglion cells from both the left and right eye project to the dorsal lateral geniculate nucleus (dLGN). The common feature characterizing this pathway is that at maturity the projections of the two eyes are segregated so that the inputs of the two eyes innervate distinct territories within the dLGN. This eye-segregated pattern arises during development from one where the inputs of the two eyes are initially overlapping. Here, I will not attempt to summarize the literature dealing with retinal waves and the formation of eye-specific projections since several recent reviews have dealt with this topic [4-6]. My intention is to challenge what I believe to be the predominant view on how segregated eye-specific projections are formed in the mammalian brain.

## Linking retinal waves to the formation of eye-specific projections

It seems reasonable to begin by asking why many people are convinced that neuronal activity plays an instructional role in the formation of eye-specific retinogeniculate projections [2,7-9]. No doubt the reasons differ for different individuals, but four factors are probably most germane to this issue. One is that in many species the developmental period when retinal waves have been found to occur spans the period when retinogeniculate projections undergo a change from an initial pattern where the projections of the two eyes are overlapping to one where these inputs are completely segregated. Such a temporal coincidence between the presence of retinal waves and the formation of eye-specific projections is an obvious requirement if these two events were to be linked causally. Second, studies that have perturbed or blocked retinal activity have often found that such manipulations prevent the formation of segregated eye-specific projections. Third, studies employing electrical stimulation of the optic nerve during the binocular overlap period have revealed that retinogeniculate projections are indeed functional during the time when segregated connections are being refined. This is an essential point since the Hebbian postulate requires that presynaptic activity be capable of activating target cells. It is also the case that convergence ratios in the retinogeniculate pathway are much higher during development than at maturity and immature synapses are functionally weaker [10], so the correlated discharges of spatially adjacent ganglion cells would seem to be an optimal means for activating lateral geniculate neurons. Finally, there is the entirely reasonable assumption that the activity patterns of the two eyes are random with respect to each other, so that at any given moment inputs converging on a geniculate cell stemming from one eye are independent of those arising from the other eye.

## Assessing the merits of the evidence

Taken together these observations appear to make a convincing case for the involvement of retinal waves in the formation of eye-specific projections. So let us briefly examine the evidence on a point-by-point basis.

### Temporal coincidence of waves and segregation of retinogeniculate projections

It is certainly the case that retinal waves have been found in a number of different species during the period when eye-specific retinogeniculate inputs are being formed (for a review, see [1]). However, in all species studied to date, retinal waves begin before and remain after the restructuring of retinal projections occurs. Thus, while there is temporal overlap in these two events there is a not a temporal congruence. Moreover, in commonly studied species such as the mouse and the ferret, the development of the visual system is compacted into a relatively brief time period, so that many of the myriad events impacting the developing visual system occur in a temporally overlapping fashion. For instance, in the mouse refinement of retinotopic projections, elimination of retinal decussation errors as well as formation of eye-specific inputs all occur during the same developmental period so distinguishing the role of retinal waves in these events can be problematic.

For this reason, several years ago we decided to assess the temporal relationship between retinal waves and the formation of segregated retinogeniculate projections in the fetal monkey, a species with a protracted developmental period [11,12]. This work revealed that the prevalence of retinal waves is greatest in the fetal monkey at embryonic day (E) 60 (gestation is about 165 days), more than a week prior to the period when retinogeniculate projections begin to become segregated. Moreover, the incidence of retinal waves was found to decrease markedly during the segregation period (E69 to E76). In follow-up work, now in progress, we have found that such a low incidence of retinal waves continues for more than two months after binocular segregation has been completed, at least until E140. The results of these studies on the fetal monkey do not rule out the possibility that retinal waves could play a role in the formation of eye-specific projections. It is curious, however, that in the fetal monkey waves are most prevalent many days prior to the time when retinogeniculate projections first begin to segregate, and relatively infrequent during the time when the projections of the two eyes are becoming segregated.

It is also the case that retinal waves have been documented in species such as the turtle, where the projections of the two eyes are entirely crossed [13]. This indicates that retinal waves can be completely dissociated from their purported role in instructing the formation of eye-specific formation. It could still be argued, however, that a seemingly ubiquitous phenomenon such as retinal waves plays an entirely different role in different species.

### Perturbation of retinal waves

A more direct way to link retinal waves with the formation of eye-specific inputs is to assess the effects of manipulations that perturb such activity. This can be achieved by various pharmacological agents or by the use of mouse mutants in which retinal activity is made to be abnormal. Initial studies relied on drugs assumed to block neuronal activity. The first study to address this issue assessed the effects of infusing tetrodotoxin (TTX) into the fetal cat brain in the vicinity of the optic tract, which presumably blocked incoming retinal activity as well as the discharges of lateral geniculate neurons [14]. This prevented the segregation of retinogeniculate projections, suggesting that neuronal activity is required for this process to occur. Since TTX can result in a high level of mortality when injected intraocularly, future studies sought a different drug to block retinal activity. Thus, Penn and colleagues [15] relied on intraocular injections of epibatadine, a cholinergic agonist, to silence retinal activity during the period in developing ferrets when cholinergic circuits drive waves. Such treatment caused retinal afferents to remain intermingled within the dorsal lateral geniculate nucleus, supporting the notion that retinal activity is essential for the formation of eye-specific projections.

These and related pharmacological studies, however, are fraught with technical and interpretative difficulties. For one thing, it is not possible to be certain that the drug in question has had the intended effect during the treatment period. While acute TTX treatment can completely block the discharges of developing retinal ganglion cells (for example, [16]), the long-term consequences of TTX treatment are unknown because it is not feasible to monitor retinal activity in the intact animal for a prolonged time period. This might explain the finding that long-term intraocular TTX injections did not prevent the formation of eye-specific retinogeniculate projections, but rather delayed their occurrence in the ferret [17]. Alternatively, the key factor may be the silencing of both pre- and postsynaptic activity, as most likely occurred in the Shatz and Stryker study [14].

To further complicate the assessment of the pharmacological studies, epibatadine, the drug reported by Penn *et al*. [15] to silence retinal ganglion cells in the developing ferret retina, has been recently shown not to block all ganglion cell discharges in either the developing ferret or mouse retina. A recent collaborative study between my laboratory and that of Barbara Chapman at UC Davis has shown that in both ferret and mouse, epibatidine decorrelates ganglion cell activity, thereby eliminating retinal waves [18]. While in both species this drug silenced the discharges of about half of all cells studied, unexpectedly, it significantly increased the activity of the remaining cells.

From the foregoing it could be concluded that it remains to be established whether blocking all retinal activity prevents the formation of eye-specific afferents, and that decorrelating retinal input perturbs the segregation of retinal projections. The latter point is challenged, however, by the finding that decorrelating retinal ganglion cell discharges by the use of an immunotoxin that eliminates cholingeric amacrine cells has no discernable effect on the normal segregation of retinal projections in the developing ferret [19].

Thus, two different approaches used to decorrelate retinal activity, epibatadine intraocular injections and cholinergic immunotoxin treatment, have different effects on the developing visual system. The former prevents the segregation of retinogeniculate projections, while the latter has no appreciable effect on the normal development of eye-specific projections. A possible resolution of this conundrum is that retinal discharge patterns following application of these drugs differ, resulting in differential effects on the developing retinogeniculate projection. Following immunotoxin treatment, the overall level of retinal activity is not appreciably different from normal, while after epibatadine treatment overall retinal activity is actually increased, in spite of the fact that this drug silences the discharges of many cells. Perhaps it is the increased firing frequency of the retinal ganglion cells that remain active after epibatamine treatment that is the critical factor in blocking eye-specific segregation. But if this were the case, it would indicate that it is not retinal waves that are crucial for the formation of eye-specific inputs, but some other feature of retinal activity.

In this context, it is worth emphasizing a point that has not been considered in the retinal waves literature. In the mouse only a relatively small proportion of ganglion cells likely project to the dLGN. This inference is based on the substantial difference between the number of ganglion cells in the mouse retina – 40,000 to 80,000 depending on the strain – and the much smaller estimate of 17,000 neurons in the dLGN [20-22]. In other species used for developmental studies, such as the cat and monkey, the number of cells in the dorsal lateral geniculate is substantially greater than the population of retinal ganglion cells [23]. Since one to three ganglion cells have been estimated to innervate a single geniculate neuron in the mature mouse [10] – a ratio not appreciably different from that in the cat and monkey – this leads to the conclusion that, in the mouse, the majority of retinal ganglion cells do not project to the dLGN. This is in line with the findings of Hofbauer and Drager [24] that a substantial majority of retinal ganglion cells in the mouse project to the superior collciulus. For this reason, it cannot be assumed that the ganglion cells impacted by a pharmacological treatment of the retina form a component of the retinogeniculate pathway. Indeed, there is a high probability that a substantial proportion of retinal ganglion cells affected by a given drug treatment do not send axons to the thalamus, and in some cases, it may well be the case that none of the ganglion cells whose activity has been impacted by a drug project to the geniculate.

Virtually all of the pharmacological studies discussed above also assume that the effects on the retinogeniculate pathway observed after blockade or perturbation of retinal activity can be explained by the Hebbian postulate. Indeed, the modeling papers that have been published in this field accept this premise as a given (for example, [25,26]). This ignores the extensive literature documenting that neuronal activity, and the subsequent calcium influx into the cytoplasm, activates a cascade of genes that play an essential role in the ingrowth and navigation of growth cones as well as the machinery required for normal synapse formation (for reviews, see [27,28]). In this light, the results of the published studies relying on drugs to perturb neuronal activity could reflect a perturbation of normal axonal growth rather than an activity-based competitive mechanism. Thus, studies that have shown an increased retinogeniculate input from the non-treated eye, after blocking activity in the other eye, could reflect an impairment of the cellular events required for normal growth and proliferation of inputs stemming from the treated eye. Under such circumstances the axons of the non-treated eye would tend to occupy expanded territory in the geniculate. This could be considered as evidence for competitive interactions between the early projections of the two eyes, but it would not reflect a Hebbian type competition. This is essentially equivalent to the expanded retinogeniculate projection that was first demonstrated in the fetal monkey and cat after *in utero *monocular enucleation [29,30].

Based on the foregoing considerations, it would seem prudent to conclude that relying on pharmacological treatments is unlikely to produce clear-cut insights into the role of activity in the formation of segregated eye-specific projections.

### Genetic manipulations of retinal activity patterns

The other means of manipulating retinal activity is by genetic mutations where selective cellular features underlying normal retinal activity are altered. To date, a variety of mutant mice have been introduced into this field and new insights have been claimed using such animals. While the results of such studies on the surface appear substantially more solid than the pharmacological treatments discussed above, a more thorough assessment reveals that such work is not without its own perils. A case in point is the literature dealing with mice lacking the β2 subunit of the nicotinic acetylcholine receptor. A number of studies have shown that retinal inputs are abnormal in these mutants; in particular, eye-specific retinogeniculate inputs do not develop normally [31-34]. This has been interpreted as supporting the role of retinal waves in the formation of segregated retinal projections since both calcium imaging studies as well as multi-array recordings reported a lack of retinal waves of the β2 knockouts [35-37].

Several years ago we obtained the two β2-/- mouse lines that have been utilized in this field with the goal of studying the formation of retinal circuitry in these animals. To our surprise, when we made multi-electrode recordings from either mutant, we found that these animals manifested robust retinal waves [38], contrary to what was previously reported. A detailed comparison of the retinal activity in the two β2-/- mice with those of wild-type animals did reveal significant differences in several parameters, but every retina from which recordings were made showed robust retinal waves. Unlike in the wild-type mouse, gap junctions rather than cholinergic synapses were found to propagate retinal waves in the mutants. It is also the case that the distribution of cells in the mutants that fired at a rate greater than 10 Hz, while lower than normal, overlapped those found in the wild-type mice. Thus, high frequency bursts are unlikely to be the driving force for eye-specific retinogeniculate projections as has been suggested by [39].

These results clearly contradict the claim that the aberrant binocular segregation pattern exhibited by the β2 mutants reflects a lack of retinal waves. Why previous studies failed to find such correlated retinal activity in these knockouts is yet to be established. The disparity between our results and those reported previously would seem to argue that recordings obtained from the isolated retina might depend more on conditions specific to a given laboratory than on real biology.

Taken all together, the available evidence indicates that perturbing correlated retinal activity does not necessarily perturb the formation of segregated retinogeniculate projections. And conversely, the presence of retinal waves does not necessarily result in the formation of segregated retinogeniculate projections.

## Some important caveats

### Acknowledging limitations of the isolated retina preparation

The β2-/- studies should remind everyone of one important caveat: the multi-electrode recordings that have been made by all laboratories to date assume that the activity patterns in the isolated retina reflect what occurs in the intact retina of the developing animal. But to my knowledge, no one has recorded retinal waves from the intact retina in a developing animal of any species. Lamberto Maffei and his then student Lucia Galli (now Galli-Resta) popularized the developing retina recording business by making ganglion cell recordings from intact embryonic and newborn rats with an extracellular microelectrode [40,41]. When two or more cells were encountered in a single electrode placement their spontaneous discharges were correlated. But this approach did not allow for the recording of retinal waves, nor was there any indication of the paroxysmal bursts that characterize a retinal wave in an isolated retina.

At present, the only way to record retinal waves is to use an isolated retina preparation as was first done by Meister and colleagues [42]. It is yet to be determined, however, what effect, if any, the severing of optic nerve axons in a young animal (a procedure that is essential for the isolated retina preparation) might have on the physiological state of retinal ganglion cells. So we are left with the unsettling possibility that retinal waves might be little more than an epiphenomenon. Until it becomes possible to observe retinal waves in the intact behaving animal, this cannot be ruled out unequivocally.

### Lacking knowledge of dLGN activity patterns when eye-specific projections are being formed

Another caveat that I believe needs to be acknowledged is the widely held assumption that retinal activity in the behaving developing animals is capable of depolarizing target cells in the dLGN as well as other retinorecipient nuclei. Several studies have shown that early in development, including the period when retinogeniculate projections become segregated, electrical stimulation of the optic pathway is capable of evoking action potentials in lateral geniculate neurons [43,44]. Moreover, using a novel *in vitro *preparation of neonatal mouse in which the retinas and portions of the dLGN are preserved, Mooney and colleagues [45] showed that the spontaneous discharges of retinal cells are capable of driving periodic discharge of dLGN neurons. This result is important since it provides the only available evidence in support of an essential component of the Hebbian model. At the same time, it is still an open question whether these results translate to the behaving animal with an intact visual system.

To my knowledge, only one study has actually recorded the activity of lateral geniculate neurons in developing animals. This was done in awake, behaving ferrets by Weliky and Katz [46] before eye opening, but well after eye-specific layers had already formed. They observed that the firings of neurons in different eye-specific layers were significantly correlated, with a strong contralateral bias. Moreover, the burst frequencies of cells within ON and OFF geniculate layers were similar. Neither of these observations would be predicted from retinal recordings made at equivalent ages. The findings of Weliky and Katz would seem to present a challenge to the prevalent formulations that retinal activity instructs the formation of eye-specific as well as ON and OFF layers in the ferret dLGN [47].

### Activity-based models cannot explain mature eye-specific projection patterns

Conventional activity-based models cannot explain two fundamental properties of mature retinogeniculate pathways. In animals with a laminated dLGN, all target cells in one layer of this structure are innervated by axons stemming from one or the other eye, while in rodents that lack a clearly laminated geniculate, all cells in a circumscribed region of the geniculate are innervated in an eye-specific manner. Activity-based models cannot explain such clustering of eye-specific cells into distinct layers or regions.

A related point is that the relationship of eye-specific inputs to geniculate layers is stereotypic in all species. Thus, in every normal cat that has ever been studied, the contralateral eye has always been reported to project to layer A and the ipsilateral eye to layer A1 of the dLGN, and similarly in the macaque monkey, layers 1, 4 and 6 of the geniculate are always innervated by the contralateral eye and layers 2, 3, and 5 by the ipsilateral eye.

If retinal waves instruct the formation of eye-specific projection patterns, how is one to explain the fact that the same eye always innervates a given layer in a species-specific manner? One possible answer would be the presence of a temporal difference in the arrival of axons stemming from one eye or the other, giving the earlier input a competitive advantage in the Hebbian scheme, resulting in that geniculate region or layer being eventually innervated only by the earlier arriving axons. But studies that have relied on modern anatomical tracers have shown that axons from both eyes innervate the dLGN from very early times [15,19].

An alternative explanation of the stereotypic pattern of retinogeniculate projections is the presence of molecular cues resulting in fibers initially differentially innervating different regions of the geniculate. According to retinal wave proponents, molecular cues are acknowledged to play a role in biasing retinal innervations, while correlated activity acts to refine the early pattern by eliminating the inputs of one eye and stabilizing those of the other eye. Why invoke an additional explanation – neuronal activity – when the molecular cues assumed to operate at an early stage of development could continue functioning throughout the period when eye-specific projections are being established? Indeed, it would be parsimonious to think that the same or related combination of molecular cues that instill the initial bias of eye-specific projections continue to operate at later stages of development, giving rise to the stereotypic eye-specific projection patterns evident in different species.

## What do retinal waves do?

Retinal waves are intriguing phenomena to study, and they are relatively easy to record once an operational multi-electrode array system has been set-up. Assuming that retinal waves are not merely an epiphenomenon, but indicative of what is truly happening in the intact animal, why are they present and what might they be doing?

I believe that the widely held viewpoint that retinal waves instruct the formation of eye-specific retinogeniculate projections via a Hebbian-type postulate is incorrect for all the reasons stated above. But this certainly does not rule out a role for retinal waves in the formation of other key features of the developing visual system. There is evidence that correlated retinal activity plays a role in the formation of ocular dominance columns in the visual cortex [48]. In addition, to cortical ocular dominance domains, a number of other features of the developing visual system could be refined by correlated retinal activity patterns. These include the elimination of topographic errors [37,49], and the progressive decrease in the convergence of retinal inputs onto geniculate neurons that occurs at relatively later stages of development [10].

Retinal waves could also provide an effective means of generating an influx of Ca^2+ ^into developing ganglion cells during the period when this is essential for turning on the cascade of genes regulating the growth, elongation and innervation of immature retinal axons. As indicated above, it is for this reason that the results of experiments that involve blocking or perturbing retinal activity are difficult to interpret from a strictly activated-mediated perspective.

Taken all together, the available evidence suggests that activity does play a role in the formation of segregated eye-specific retinogenioculate projections, but not by means of a Hebbian competitive model. As noted above, a plausible hypothesis that needs to be rigorously tested is that the presence of normal activity patterns is required for the normal growth and elaboration of retinal axons. It is also likely, but still unproven, that retinal activity regulates the expression of molecular cues within target nuclei that underlie the formation of eye specific projections. In my view, there is bound to be a synergistic relationship between neuronal activity and molecular cues, so that both factors interact throughout development, leading to the formation of eye-specific inputs. Studies of the type carried out to explain formation of topographic maps [50,51] where ephrin signaling and retinal activity have been shown to be linked, offer the best opportunity to obtain the correct picture of how eye-specific retinogeniculate inputs are formed. To date, several papers have been published implicating the expression of molecular cues in this developmental process [52-54]. While these offer a promising start, the discovery of eye-specific molecules and their regulation by retinal waves of activity is yet in the future. When it becomes recognized that retinal waves do not instruct the formation of eye-specific retinogeniculate projections, a major step forward will have been taken in unraveling the true role of retinal activity in this fascinating developmental story.

## Abbreviations

dLGN: dorsal lateral geniculate nucleus; E: embryonic day; TTX: tetrodotoxin.

## Competing interests

The author declares he has no competing interests.
